# SARS-CoV-2 COVID-19 susceptibility and lung inflammatory storm by smoking and vaping

**DOI:** 10.1186/s12950-020-00250-8

**Published:** 2020-06-10

**Authors:** Gagandeep Kaur, Giuseppe Lungarella, Irfan Rahman

**Affiliations:** 1 Department of Environmental Medicine, University of Rochester Medical Center, Box 850, 601 Elmwood Avenue, Rochester, NY 14642 USA; 2 Department of Molecular and Developmental Medicine, University of Siena, Siena, Italy

**Keywords:** COVID-19, ACE2, SARS-CoV2, Smoking, Vaping, E-cigarettes, Inflammation

## Abstract

The current pandemic of COVID-19 has caused severe morbidity and mortality across the globe. People with a smoking history have severe disease outcomes by COVID-19 infection. Epidemiological studies show that old age and pre-existing disease conditions (hypertension and diabetes) result in severe disease outcome and mortality amongst COVID-19 patients. Evidences suggest that the S1 domain of the SARS-CoV-2 (causative agent of COVID-19) membrane spike has a high affinity towards the *angiotensin-converting enzyme 2* (ACE2) receptor found on the host’s lung epithelium. Likewise, TMPRSS2 protease has been shown to be crucial for viral activation thus facilitating the viral engulfment. The viral entry has been shown to cause ‘cytokine storm’ involving excessive production of pro-inflammatory cytokines/chemokines including IL-6, TNF-α, IFN-γ, IL-2, IL-7, IP-10, MCP-3 or GM-CSF, which is augmented by smoking. Future research could target these inflammatory-immunological responses to develop effective therapy for COVID-19. This mini-review provides a consolidated account on the role of inflammation and immune responses, proteases, and epithelial permeability by smoking and vaping during SARS-CoV2 infection with future directions of research, and provides a list of the potential targets for therapies particularly controlling cytokine storms in the lung.

## Introduction

What started with symptoms of pneumonia of unknown etiology in December 2019 from the Wuhan city district in China, has now become a global health concern and declared as pandemic. The causative agent of COVID-19, SARS-CoV-2, is not an unknown pathogen. There have been two previous outbreaks caused by members from the same family (i.e., *Coronaviridae*) of RNA virus, i.e., SARS and MERS [1]. However, the current SARS-CoV2 is highly infectious with a higher tendency of human-to-human transmission [2, 3]. Considering that there exists no known cure for this infection, rapid screening/testing, tracing and containment are the most effective means to fight the pandemic at present. This makes it crucial to identify the susceptible groups who are at a higher risk for complications. Some of the cases of SARS-CoV2 infection leading to acute respiratory distress syndrome (ARDS) and severe disease outcome have been reported amongst people with old age (senescence with dampened immunity) or co-morbidity [4]. Epidemiological data show that the elderly and individuals suffering from pre-existing diseases (like diabetes or hypertension) have a higher mortality rate amongst patients with COVID-19, while young and healthy adults show a different disease tropism [5]. Additionally, there are a number of patients who are at a higher risk of contracting the disease, for instance patients with cancer, transplants, or other conditions requiring the use of immune-suppressing drugs especially with respiratory illnesses [6]. In particular, the disease, (a) may not manifest itself at all; making the patient a potential carrier of the viral pathogen, (b) may manifest as “banal flu” thus being undiagnosed till further complications, and (c) may develop into a serious illness requiring hospitalization. This makes it crucial to understand the exact mode of disease development and progression, and identify the population group that might be at a higher risk of contracting the disease. In this respect, people with a smoking/vaping history may be at a higher risk of suffering from severe disease outcomes by COVID-19 infection. However, at present there is little or no significant data available regarding the health implications towards SARS-CoV-2 infection in smokers/vapers. In this mini-review, we present the evidences that tobacco smoking and/or vaping e-cigarettes could weaken the lungs defenses, thus rendering the individuals more susceptible towards COVID-19 infection with augmented symptoms.

## COVID-19 and smoking/vaping

Though there is no direct evidence suggesting the increased susceptibility of smokers/vapers towards COVID-19 infection, various indirect studies prove that this population is at a higher risk to show severe symptoms and need mechanical ventilation as compared to non-smokers. While analyzing the factors associated with severe disease outcomes in patients admitted to three tertiary hospitals in the Wuhan district, Liu et al (2020), showed that patients with a history of smoking were significantly higher in the progression group (with severe symptoms) than improvement (patients showing recovery) group (27.3% vs 3%) [4]. Again, in Wuhan, the epicenter of the disease in China, the case fatality rates amongst males (2.8%) has been reported to be higher than females (1.7%). Similar trends have been reported in other hard-hit regions like Italy and Spain, where out of several cases of COVID-19 infection, 58% were men. Furthermore, men are more prone to succumb to the disease with fatalities (72%) [7]. One of the prime reasons for this has been speculated to be higher rate of smoking amongst men (52.1%) than women (2.7%) [8, 9]. Further, smokers are more prone to contract respiratory infections with higher rates of influenza, tuberculosis and pneumonia than non-smokers [9]; which supports the rationale of considering this population group to be at high risk.

## Role of epithelial cells and inflammatory response: ACE2 and TMPSSR2

The study of the pathogenesis of COVID-19 points towards the dangers of contracting a disease and eventual complications amongst smokers/vapers due to delayed clearance of virus.

SARS-CoV2 belongs to the family of Coronavirus that obtains its name from the crown-like appearance on imaging. This feature is attributed to a glycosylated cell surface spike (S) protein with two functional domains-S1 and S2. ACE2 has been shown to be the site of host-cell entry for the SARS-CoV2 virus. The S2 domain of the viral spiked envelope has a high affinity to the ACE2 receptor on the lung epithelium. Interestingly, ACE2 expression has been found to be high amongst smokers (possibly including e-cigarette vapers) and individuals on ACE blockers (patients with hypertension and diabetes), thus rendering them susceptible to the disease [9]. Furthermore, there are more circulating ACE2 in men which provides evidence for gender-based variations in disease severity [10]. ACE2 may be highly expressed in germ cells i.e. more in men vs women. It is likely that ACE2 is related to *nicotinic acetylcholine receptors* (nAChRs), particularly alpha7nAChR receptor further supporting that smoking/vaping (nicotine) status might be crucial in the pathophysiology of COVID-19 [11]. The ACE2 receptors (developmentally regulated) are abundant on the lung epithelium, specifically the type II pneumocytes, goblet, nasal epithelial/ciliated and oral mucosal cells [12–14]. A recent study has suggested a role of interferon-stimulated response of SARS-CoV-2 entry via ACE2 and TMPSSR2 protease [15]. Studies suggest that ACE2 expression is upregulated in the small airway epithelia of smokers and patients with smoking-associated pathologies like COPD and IPF [15, 16]. Though not tested, vaping (nicotine) may have similar effects, thus making this group more prone to be affected by the disease.

While ACE2 is important for host entry, the host cellular proteases function to activate the viral particle thus facilitating the viral engulfment. In this respect, TMPRSS2 protease is of importance in that ACE2 employs the cellular serine protease TMPRSS2 for S protein priming and host-cell entry [17]. Studies show that the SARS-CoV-2 entry-associated protease, TMPRSS2, is highly expressed in the nasal ciliated and goblet cells. Single cell RNA sequencing analyses of multiple tissues has shown that only a small subset of ACE2+ cells express TMPRSS2, thus suggesting that other proteases might play similar role. In this respect, Cathepsin B/L has also been shown to be of importance [14]. Interestingly, in vivo and clinical data show that cigarette smoke results in increased expression of Cathepsin B, which raises the possibility of increased susceptibility towards COVID-19 infection amongst smokers [16]. Another cellular protease, furin, cleaves the S1/S2 site of the spike protein of SARS-CoV-2 which is essential for the cell-cell transmission of the virus [18]. Smoking can decrease the effectiveness of serine protease inhibitors (serpins) that control the “furin” activity [19, 20]. Also, evidence suggests that serpin-deficiency attributes to increased viral (Influenza A) susceptibility in C57BL/6 mice [21]. Taken together, these findings point toward increased possibility of COVID-19 contraction amongst smokers/vapers.

**Fig. 1:**
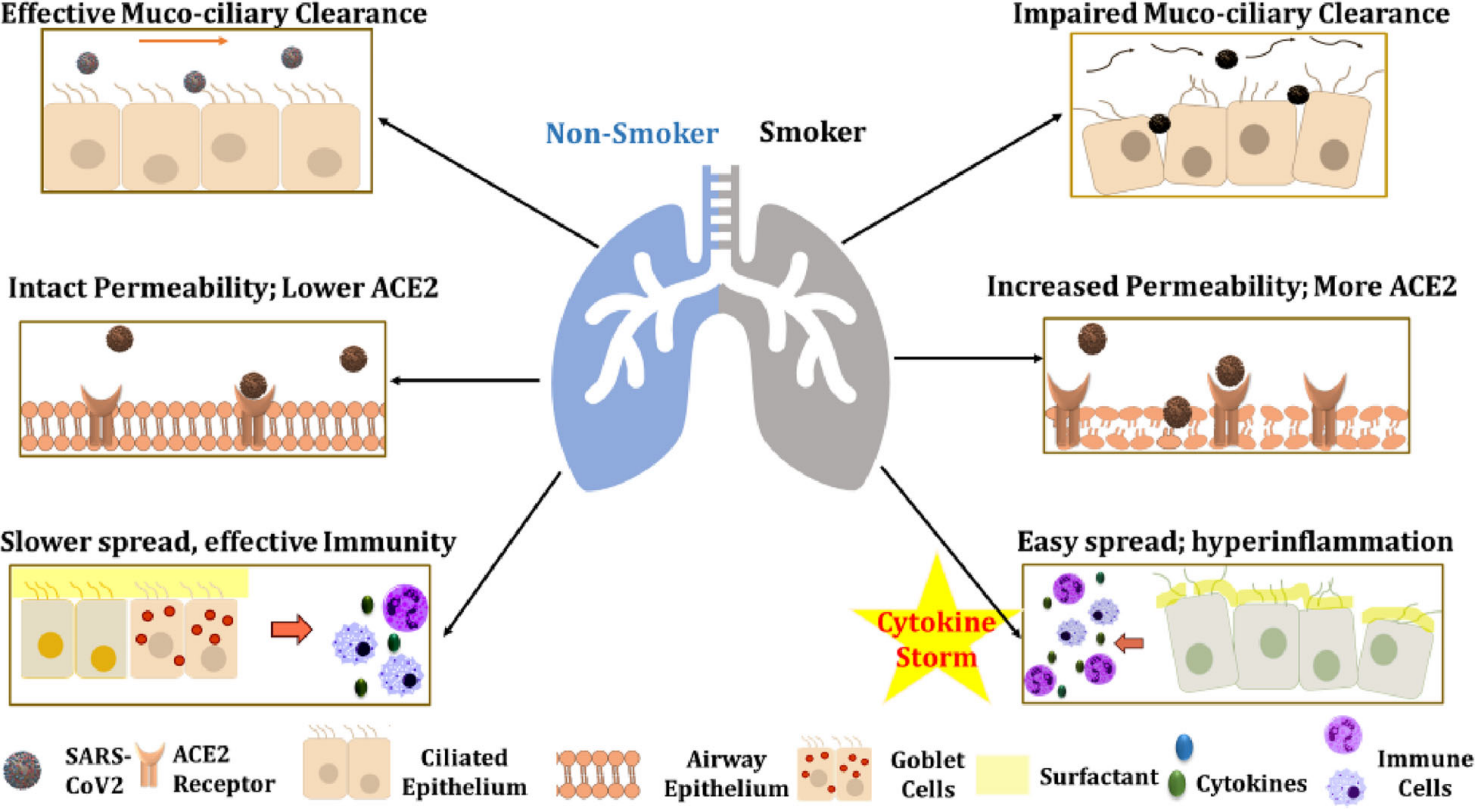
Factors responsible for higher susceptibility of smokers/vapers against COVID-19. In normal individuals, the muco-ciliary epithelium and the mucous layers act as the first line of defence against the foreign pathogen (in this case SARS-CoV2). On smoking, this layer is damaged and so is the flow of the peri-ciliary fluid (mucous; indicated by arrows) which makes them more prone to infections. Smokers are also shown to have higher surface expression of ACE2 receptors (binding sites for SARS-CoV2) which allows the entry of pathogens into the host cell and protects the virus against the host surveillance. In normal individuals, the viral infection could be checked by the, (**a**) cytokine release from the type II pneumocytes, goblet, nasal epithelial/ciliated and oral mucosal cells and (**b**) immune cell (macrophages, neutrophils and lymphocytes) infiltration at the site of infection, to contain further spread. Smoking weakens the immune system enabling easy entry into the host cell, rapid multiplication of the virus followed by hyperinflammatory response triggered by ‘cytokine storm’ in the host body eventually leading to damaged lung tissue

Smoking and vaping also affect the tight barrier junction leading to increased epithelial permeability (lung leakiness). In fact, the structural changes due to cigarette smoking including; increased mucosal permeability, impaired muco-ciliary clearance, peribronchiolar inflammation and fibrosis (airway remodeling); could pose little to no resistance towards viral entry amongst smokers as shown in Fig. 1 [22].

Smoking/vaping causes oxidative stress and inflammatory responses in the lung which make smokers/vapers more susceptible to bacterial/viral infections [23–25]. Oxidative stress has adverse effects on the epithelial permeability and ACE2 expression, which may have serious implications in smokers/vapers [26, 27]. ACE2 exists in multiple isoforms with predominance of 90 kDa in the lungs and 120 kDa in kidneys [26]. It can be post-translationally modified by oxidants/carbonyls. Hence ROS generation due to smoking or vaping could adversely affect the ACE2/Angiotensin (1-7) /Mas axis [28]. Likewise, the oxidative stress due to cigarette smoke or e-cig aerosols results in epithelial barrier dysfunction which increases the membrane permeability and susceptibility towards viral/bacterial infections [28–30] (Fig. 1). Covid-19 related genes, such as ACE2 and TMPRSS2 are affected in patients with asthma and are linked with inhaled corticosteroids [31]. This suggests that steroid-resistance seen by smoking in patients with COPD may have ramifications in COVID-19 susceptibility via ACE2 and TMPRSS2.

The most common complication due to SARS-CoV2 infection -Acute respiratory Distress syndrome (ARDS) - is a result of the ‘cytokine storm’ caused due to uncontrolled release of proinflammatory cytokines/chemokines by effector immune cells [32]. These pro-inflammatory mediators include IP-10, MCP-3, HGF, MIG, MIP-1α, IL-6, TNF-α, IFN-γ, IL-2, IL-7 and GM-CSF. In fact, while studying the transcriptional response towards infection using in vitro model, Blanco-Melo et al. found that SARS-CoV-2 infection in normal human bronchial epithelial cells results in reduced IFN-mediated responses along with heightened production of cytokines/chemokines that enables sustained viral replication [33]. Of interest, the expression of IL-6, TNF-α and other pro-inflammatory cytokines is upregulated in chronic smoking condition and so is the low expression levels of perforin and granzyme B- the two major effector proteins of natural killer (NK) and CD8 T cells [34]. Furthermore, lung autopsy of COVID-19 patients demonstrated neutrophil infiltration in pulmonary capillaries with fibrin deposition and extravasation of neutrophils into the alveolar space [35]. These observations point towards formation of Neutrophil Extracellular Traps (NETs) that may contribute to organ damage, lung remodelling, and mortality in COVID-19 patients. Evidence suggests that smoking affects neutrophil trafficking, NET formation, humoral and cell-mediated immune responses as shown in Fig. 1. This could eventually lead to susceptibility towards ARDS development further augmenting the disease pathogenesis [35].

In this respect, it is important to mention about the susceptibilities of old smokers/vapers with comorbidities like COPD or IPF. It is known that older patients are more likely to develop pneumonia and respiratory failure due to SARS-CoV-2 infection, which suggests that cellular senescence might play an important role in the disease pathogenesis for COVID-19 [35]. It has been shown that a lowered expression of regulatory proteins like TRIB3 (negative regulator of NF-kappaB signaling) and SIRT1 (anti-aging and anti-inflammatory) amongst aged/older individuals, rendering them more prone to infection [36, 37]. Interestingly, our group showed that the SIRT1 expression is lowered in the lungs of smokers and COPD patients [37]. This highlights the possible involvement of SIRT1 and senescence-associated pathways in modulating SARS-CoV-2 pathogenesis in humans, thereby putting smokers/vapers at a higher risk of contracting an infection. Further, gene tribbles homolog 3 (TRIB3) is decreased during aging in male, and its protein interacts with nucleocapsid protein and RNA dependent polymerase of the virus [36].

The same could be the case with vapers who use e-cigarettes. Considering the rapidly increasing cases of vaping-induced lung pathologies, CDC has coined the term ‘e-cigarette or vaping product use associated lung injury (EVALI)’ to characterize conditions like acute lung injury, acute fibrinous pneumonitis, diffuse alveolar damage, or pneumonia accompanied by bronchiolitis. Such insults increase the possible risk amongst vapers. In contrast, e-liquid constituents interact with the pulmonary surfactants, i.e. dipalmitoylphosphatidylcholine (DPPC), which could lead to induction of innate immune responses in ENDS users. The lipid dysregulation in the airways of ENDS user could render them more prone to the COVID-19 infection [38]. Furthermore, it is possible that EVALI and COVID-19 have similarities as both develop interstitial pneumonia leading to ARDS [38]. In fact, both EVALI and COVID-19 are characterized by decreased arterial oxygen saturation and bilateral pneumonia, thus making it difficult in detecting the two conditions [39, 40] Also, considering that many people with COVID-19 infection do not show any symptom of the disease, there is a likelihood of the vapers with EVALI being the asymptomatic carriers of COVID-19 [41]. Further, research is required to test the hypothesis that COVID-19 and vaping are associated with severe EVALI. Similar to EVALI, patients with COVID-19 showed elevated levels of several cytokines (CCL2/MCP-1, CXCL10/IP-10, CCL3/MIP-1A, and CCL4/MIP1B) in BALF and peripheral blood mononuclear cells [42].

It is also possible that exosomes/extracellular vesicles released by lung epithelial cells may trap SARS-CoV2 and their respective miRNA/RNA, which would trigger a cytokine storm to other neighboring cells in response to smoking [43]. ACE2 positive cells may play a role in viral entry [44]. Exosomes may be pro-inflammatory or can be exploited pharmacologically in lung diseases [45, 46].

## Therapeutic targets and approaches

**Fig. 2:**
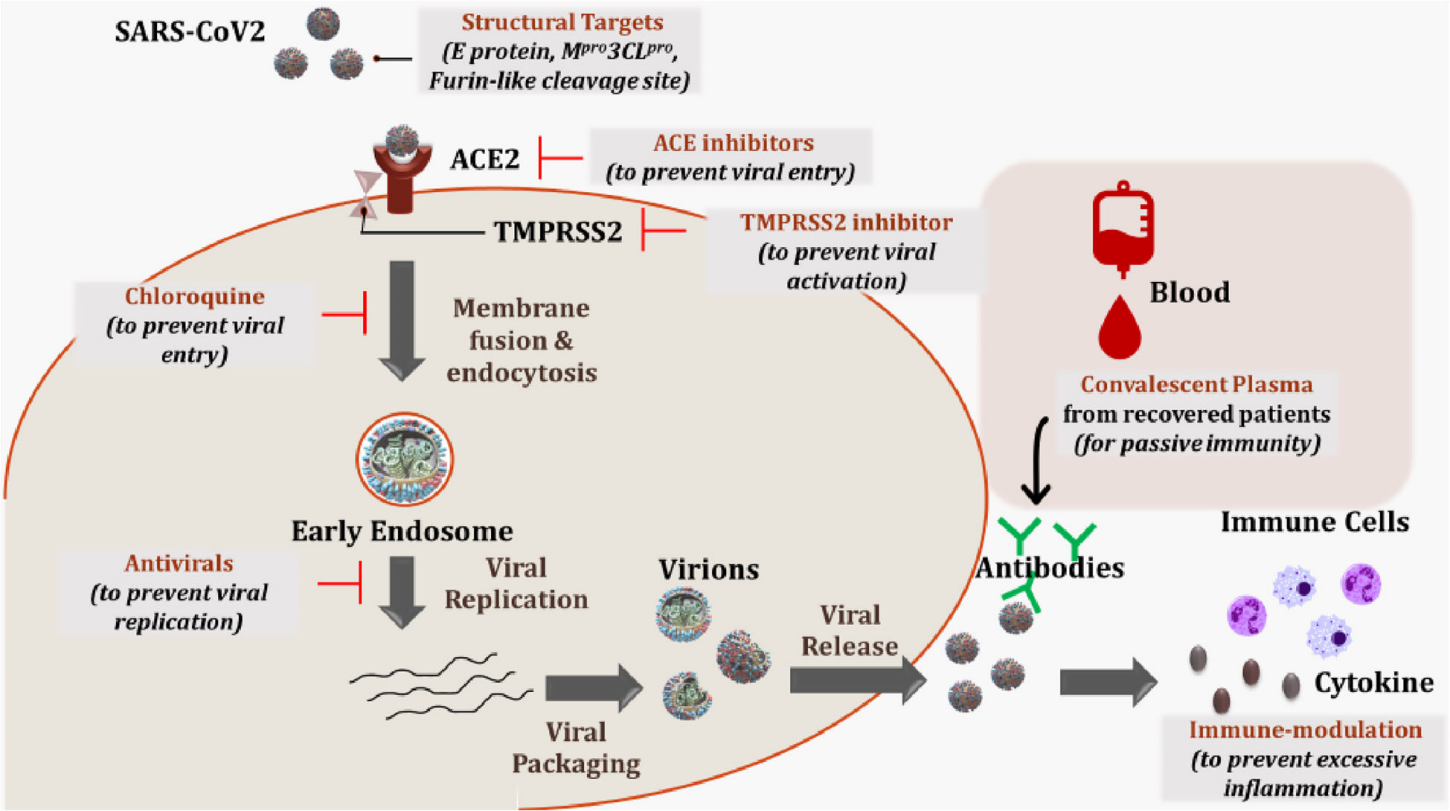
Potential drug targets and strategies being tested to contain SARS-CoV-2 infection. SARS-CoV2 has a high binding affinity to ACE2 receptors found in abundance in the type II pneumocytes in the lung alveolar epithelium. This could be targeted by ACE inhibitors/ARBs. On binding to the ACE2 receptors, TMPRSS2 protease cleaves the viral S protein and enable the engulfment of the viral body into the cell. Here the viral proteases enable the fusion of the endosomal membrane and release of viral RNA (genome) into the host’s cytoplasm. TMPRSS2 inhibitors or antimalarial drugs (chloroquine/hydroxychloroquine) can prevent this engulfment. The viral RNA now hijacks the host’s translational machinery to express viral proteins and further enables viral replication and packaging. This is the site of action for various antiviral drugs being tested in clinical trials at present. The viral RNA and protein get packaged as virions which are ultimately released into the extracellular space, where it leads to release of pro-inflammatory cytokines/chemokines and infiltration of immune cells. It is speculated that the heightened immune response is responsible for the disease severity in COVID-19 patients, thus immune-modulation is one of the alternate to be used to prevent serious outcomes. Alternate approaches like targeting viral structural targets or use of convalescent plasma to induce passive immunity are other strategies which are being explored at present

Currently, there is no known treatment or vaccine for the disease and there is limited knowledge about the disease pathogenesis. Several efforts are being made to design a drug or vaccine against SARS-CoV2 infection [47]. We reviewed some of these approaches based on mechanism of action in Table 1 listing their pros and cons. Also, a diagrammatic representation of the viral entry into the host and potential sites of modulating its antigenicity has been depicted in Fig. 2. These approaches can be used alone or in combinations to combat against the virus.

**Table 1:** Therapeutic options for COVID-19 with respect to lung cytokine storms by COVID-19

Treatment/ Prophylaxis Options	Rationale	Pros	Cons	Reference
***Targets from host’s immune system*** ***(IL6 blockers: Actemra, Tocilizumab.*** ***Monoclonal antibodies: Kevzara-sarilumab)***	Preventing excessive inflammatory responses.	Modulation of host’s immunopathological responses would decrease risks of ARDS.	Immune-modulation could have adverse effects	[48]
***ACE inhibitors (*e.g.*Umifenovir)***	ACE inhibitors may target the S1 domain and ACE2 interaction thus preventing virus entry.	ACE modulation has successfully been employed in treating conditions like hypertension, heart failure and atherosclerosis.	Role of ACE inhibitors in COVID-19 is not clear.	[9, 49]
***TMPRSS2 inhibitor (*e.g.*Camostat, Nafamostat)***	TMPRSS2 inhibition could prevent the viral activation preventing virus entry.	TMPRSS2 inhibition may have little on-target side effect.	Proteases other than TMPRSS2 (e.g. Cathepsin L, TTSP) might have a role in viral activation.	[50, 51]
***Chloroquine/Hydroxychloroquine***	It could prevent viral entry and have other immune-modulatory effects.	Its anti-inflammatory properties could help monitor immunopathological responses in patients.	It is associated with side effects like nausea, headache, blurred vision, vomiting, cramps, and diarrhea.	[52–54]
***Anti-viral agents (*e.g.*remdesivir, favipiravir, ripavirin)***	They target the viral replication by inhibiting the RNA polymerase enzyme.	These agents have shown promising results during initial clinical trials.	Use of antivirals has the risk of developing resistance amongst some patient populations.	[47, 55]
***Convalescent Plasma***	It involves use of passive antibody therapy to provide viral neutralization.	It can be used for both prophylaxis or treatment. Its efficacy has been tested in previous infections like SARS, Ebola, and hepatitis.	It has a known risk of inadvertent infection due to blood transfer and antibody dependent enhancement of infection (ADE).	[56]
***Targets from viral structure (like; E protein, M***^***pro***^***3CL***^***pro***^***, Furin-like cleavage site)***	Targeting viral structure would prevent viral host-cell entry and replication.	This method would design SARS-CoV2-specific treatment	Finding a potential cure by this method might take some time before it reaches clinic.	[57–59]

The most important target that has emerged so far is, ACE2. It is the site of attachment of the virus to the host’s cell and the first port of entry for the virus which may be affected by smoking in lung epithelial cells. Consequently, targeting ACE receptor binding is the most popular strategy of SARS-CoV2 containment [9]. However, ACE2 is a key modulator of renin-angiotensin system and it is not clear how its inhibition functions in patients with COVID-19. It is thought that administering these drugs might increase the disease severity. Also, due to ACE2 polymorphism or isoforms, some individuals might be genetically predisposed to the disease which could be hard to monitor using this approach [49]. Overall, currently there is no clinical evidence that proves the efficacy of angiotensin-converting enzyme (ACE) inhibitors or angiotensin II type 1 receptor blockers (ARBs) in treating COVID-19 infection [60].

While ACE2 binds to the viral S2 domain, there are various membrane proteases that function to activate the internalization of the virus into the host cell. The TMPRSS2, in particular, has been shown to be crucial in this respect. In fact, TMPRSS2-mediated viral activation and entry has been reported for other coronaviruses and influenza virus [50]. In vitro studies have found that blocking of TMPRSS2 activity using, camostat mesylate, results in the partial inhibition of the SARS-CoV2 entry into the lung epithelial cells, making it a potential target for drug development [17]. On the downside, however, reports suggest that TMPRSS2 is not solely responsible for host cell entry in case of coronaviruses. It seems that many other proteases (Cathepsin L, TTSP family members) could serve similar purpose [51].

Antimalarial drugs, chloroquine and hydroxychloroquine, are also being tested against SARS-CoV2 infection. Clinical trials using hydroxychloroquine have been planned due to the endorsements and proven relative safety for the effectiveness of this drug for COVID-19. Early clinical studies support the efficacy of hydroxy−/chloroquine in reducing viral load and shortening the disease duration, but such studies are small, anecdotal, and non-robust [52–54, 61].

Targeting the SARS-CoV2 structure could be a successful strategy to prevent viral infection. In this respect, many groups have identified some potential viral structural targets like the SARS-CoV2 protease, protein E or furin-like S1/S2 cleavage site, and RNA polymerases [57–59]. Nevertheless, research is in progress to understand the structural uniqueness of this virus and developing a potential drug using this approach might take some time.

Antiviral drugs (like, remdesivir and favipiravir) are another group of drugs that are currently under clinical trials and are showing promising results [62, 63]. These drugs have varying modes of action. For instance, remdesivir is an adenosine analog that inhibits the enzyme RNA polymerase thus preventing viral replication. Similarly, favipiravir is RNA polymerase mutating agent [55]. An alternate approach to prevent viral spread could be to target the host’s immune response. Patients with COVID-19 have been shown to suffer from a cytokine storm characterized by increased levels of IL-6 [47]. While studying the host-immune response against the virus using in vitro model, lowered innate antiviral defences coupled with exuberant inflammatory cytokine production was demonstrated. Considering this, immune-modulation techniques targeting IL-6 binding or IFN-1 signaling could be a suitable remedy to protect against infection [33]. Similarly, HLA haplotypes are known to be responsible for varied susceptibilities towards infections in human. CD4^+^ or CD8^+^ T cells recognize the viral antigens to wage an immune response. Studying the HLA loci associated with development of anti-SARS-CoV-2 immunity for induction of protective immunity could be a potent way to combat against the virus [64]. However, such studies need to be performed with caution, as immune-modulation might have serious side-effects [65]. Lastly, the use of convalescent sera is being tested by several pharmaceutical companies to generate antibody preparations against SARS-CoV2 from convalescent plasma of recovered patients; but there are risks associated with its use as discussed in the Table 1 [56].

## Conclusions and future directions

In conclusion, a better identification of susceptible individuals (particularly with smoking and vaping histories) and their early detection, as well as targeting ACE2 receptor (and isoforms), surface proteases, and other immunological/inflammatory targets could be a major game changer in managing the spread of this pandemic and further infections. Although many questions still remain unanswered which need to be considered for future research. Considering that many smokers/ex-smokers with airway obstructions show steroid resistance (e.g., severe asthmatics and patients with COPD), the use of steroids for management of COVID-19 must be reconsidered. It is not clear if the disease severity arises due to excessive production of pro-inflammatory responses or reduction in anti-inflammatory mediators (proresolving mediators), affecting alveolar inflammation, and due to secretory associated senescence phenotype (SASP) in aging lungs. Senesced cells may harbor components of RNA or RNA virus may hijack the host cells, and release exosomes/extracellular vesicles containing pro-inflammatory miRNA and/or viral genome/spike protein/ACE2 receptor. Careful examination of the macrophage responses on COVID-19 infection with the assessment of the phenotypic switching might be of interest in future. Further studies are required to determine the contribution of NETs in alveolar inflammation, role of epithelial permeability, and proteases in SARS-Cov2 epithelial entry. Also, the health implications of this infection amongst the recovered population remains unknown (e.g. will they develop more recurrent infections and lung remodeling/impaired repair leading to pulmonary fibrosis over a period of time). Given the ACE2 polymorphism, the role of genetic and epigenetic factors in governing the disease susceptibility (particularly in patients with hypertension) is yet another consideration to guide future research. Overall, understanding the mechanisms involved in the pathogenesis of SARS-CoV2 infection with subsequent pneumonia with lung cytokine storms, and identifying the emerging therapeutic strategies will provide timely treatment of this devastating disease.

## Data Availability

Not applicable. No data are available as this is a review article.

## Abbreviations

COVID-19: Coronavirus Disease 2019
SARS-CoV2: Severe Acute Respiratory Syndrome Coronavirus 2
ACE2: Angiotensin-converting enzyme 2
ARDS: Acute Respiratory Distress Syndrome
COPD: Chronic Obstructive Pulmonary Disease
SARS: Severe Acute Respiratory Syndrome
MERS: Middle East Respiratory Syndrome
TMPRSS2: Transmembrane protease serine 2
TTSP: Type 2 Transmembrane Serine Proteases
NET: Neutrophil Extracellular Traps
HLA: Human Leukocyte Antigen
